# Supplementary material for: Data-driven analysis of amino acid change dynamics timely reveals SARS-CoV-2 variant emergence
Source: Sci Rep. 2021 Oct 26;11:21068. doi: 10.1038/s41598-021-00496-z (PMC8548498; doi:10.1038/s41598-021-00496-z)
Supplement: Supplementary file 1 — Supplementary Information. [file 41598_2021_496_MOESM1_ESM.pdf]

# Supplementary Materials

## Data-driven analysis of amino acid change dynamics timely reveals SARS-CoV-2 variant emergence

**Anna Bernasconi[1,*], Lorenzo Mari[1], Renato Casagrandi[1], and Stefano Ceri[1]**

[1]Politecnico di Milano, Departement of Electronics, Information, and Bioengineering, Milan, 20133, Italy
[*]anna.bernasconi@polimi.it

## Supplementary Figures

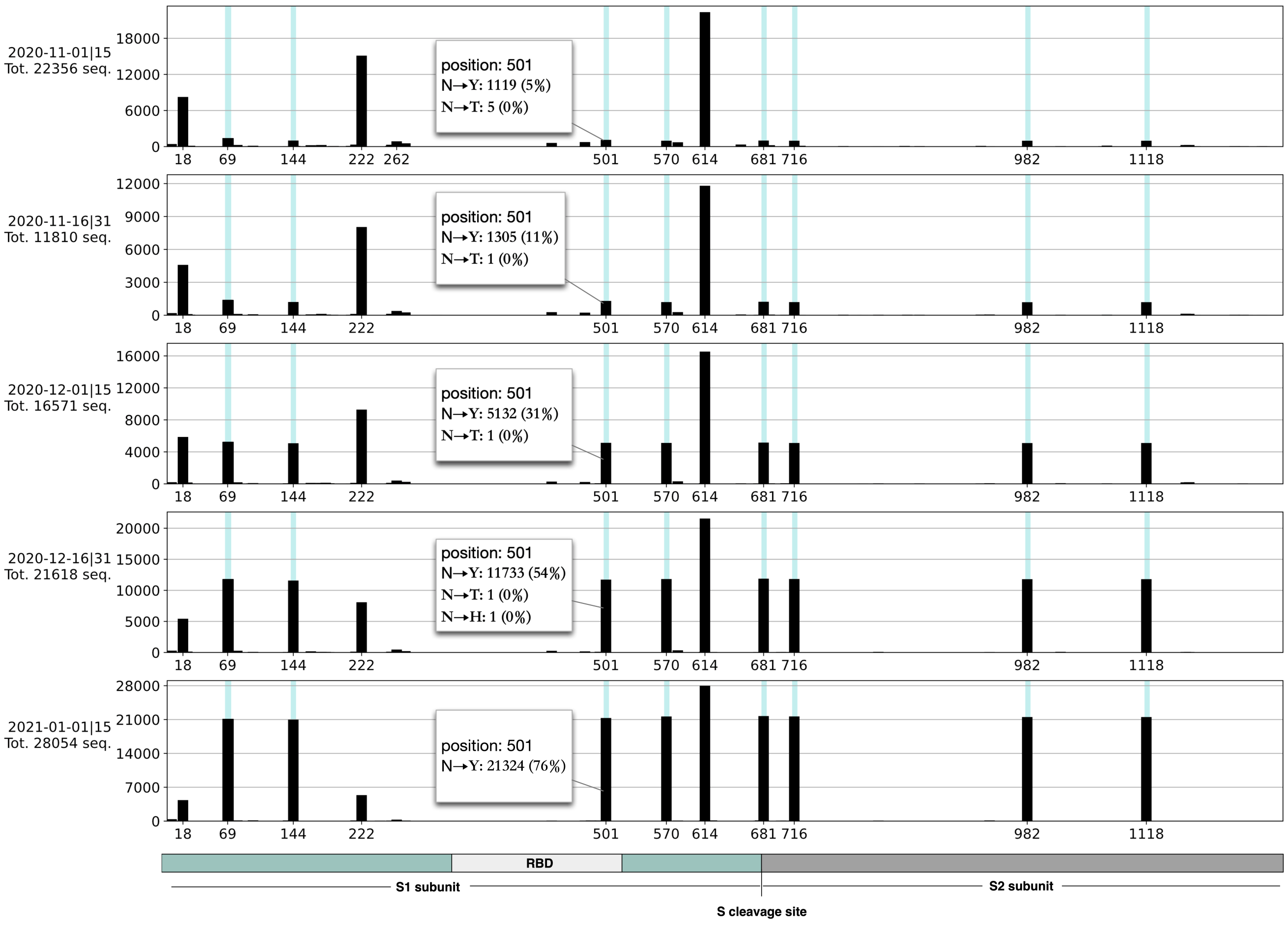


**Figure S1.** Counts of protein Spike amino acid changes in the UK in different bi-weekly periods. The tracks represent sequences of the five groups filtered by date (from the first half of November 2020 till the first half of January 2021). A blue background is used in the positions notably belonging to the Alpha variant, as first recognized as the B.1.1.7 lineage by Rambaut *et al.*[50]: H69-, V70-, Y144-, N501Y, A570D, P681H, T716I, S982A, and D1118H; higher black bars in these positions from top to bottom indicate an increase in the frequency of these amino acid changes.
The plot was created using the Matplotlib library v3.3.4 of Python v3.8.5 (https://matplotlib.org/).

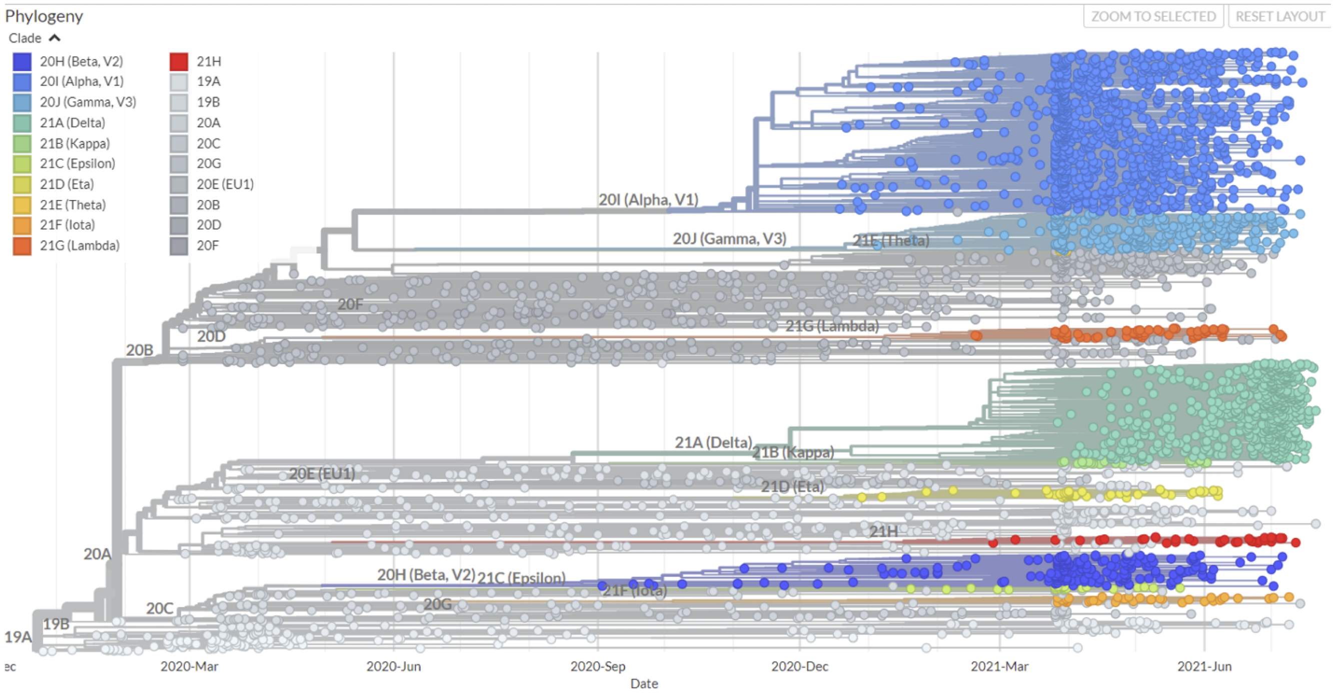


**Figure S2.** Example phylogenetic tree of SARS-CoV-2 major variants.
Image generated with Nextstrain[14] on Aug. 2, 2021 (`https://nextstrain.org/ncov/gisaid/global`).

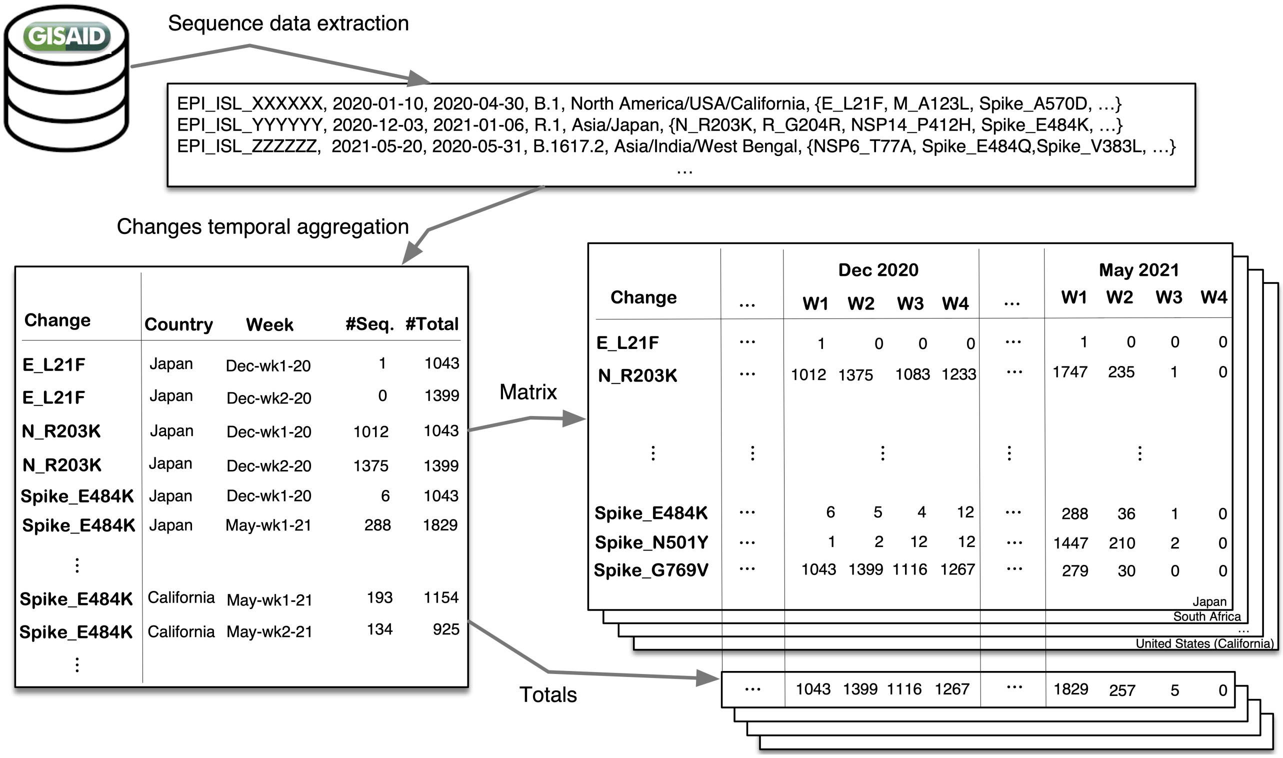


**Figure S3.** Data extraction and aggregation steps. GISAID original data comprise records of sequence accession ID, collection date, submission date, Pangolin lineage, collection location, and list of amino acid changes. We compute a table of tuples with: amino acid change, country of collection, week of collection, absolute count of sequences holding that change, and total collected sequences in the same country-week. For selected countries (the example shows Japan), we build a matrix where rows are amino acid changes observed in at least five sequences for at least three weeks, columns are observation weeks, and cells contain the number of sequences collected in the given week exhibiting the specific amino acid change. A separate vector holds the total number of sequences collected in the location under study for each of the considered weekly periods. Note that the last weeks of May 2021 hold very few data, thereby producing diluted results of our clustering and warning method. Image generated with OmniGraffle v6.6.2 (`https://www.omnigroup.com/`).

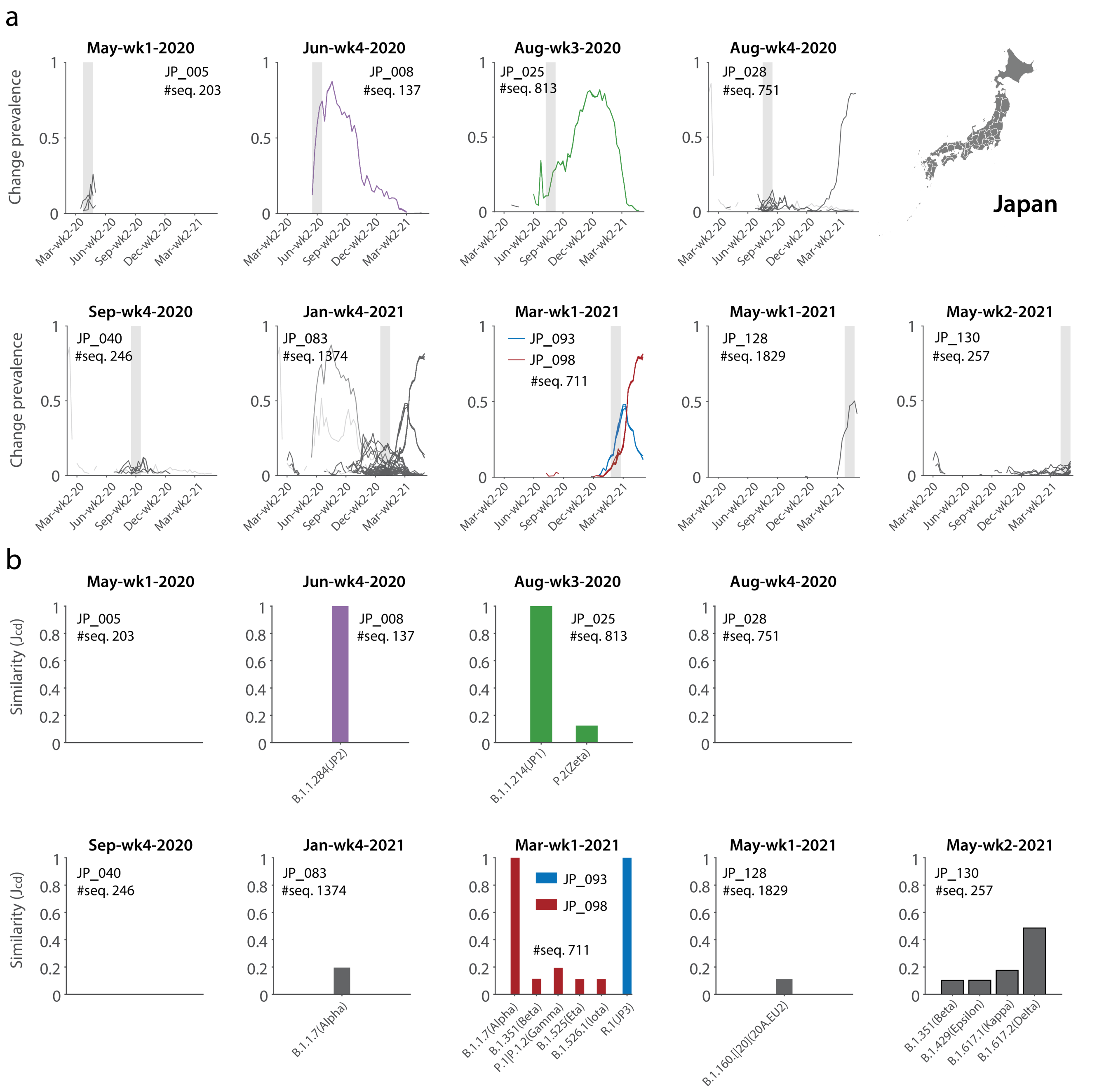


**Figure S4.** Data-driven warning of possible candidate variant emergence issued in Japan. (**a**), The nine time points when warnings were raised. Note that Mar-wk1-21 exhibits two distinct warnings for clusters JP_093 and JP_98. (**b**), Cluster-lineage Jaccard similarity for the ten warnings. The four hits (similarity above the threshold $J_{cd} = 0.5$) are reported in Fig. 1 in the main text. Strong cluster-lineage similarity is already observed at the time when clustering is performed, which qualifies these as early hits. In this case, candidates were discarded within five weeks, as no late hits (i.e. strong similarity observed with some delay after clustering/warning between a cluster with an amino acid change composition that can be traced back to the original warning and a lineage dictionary) were recorded.

Plots were created using MATLAB R2021a (http://www.mathworks.com). All graphics were further processed using Adobe Illustrator 2021 (http://www.adobe.com).

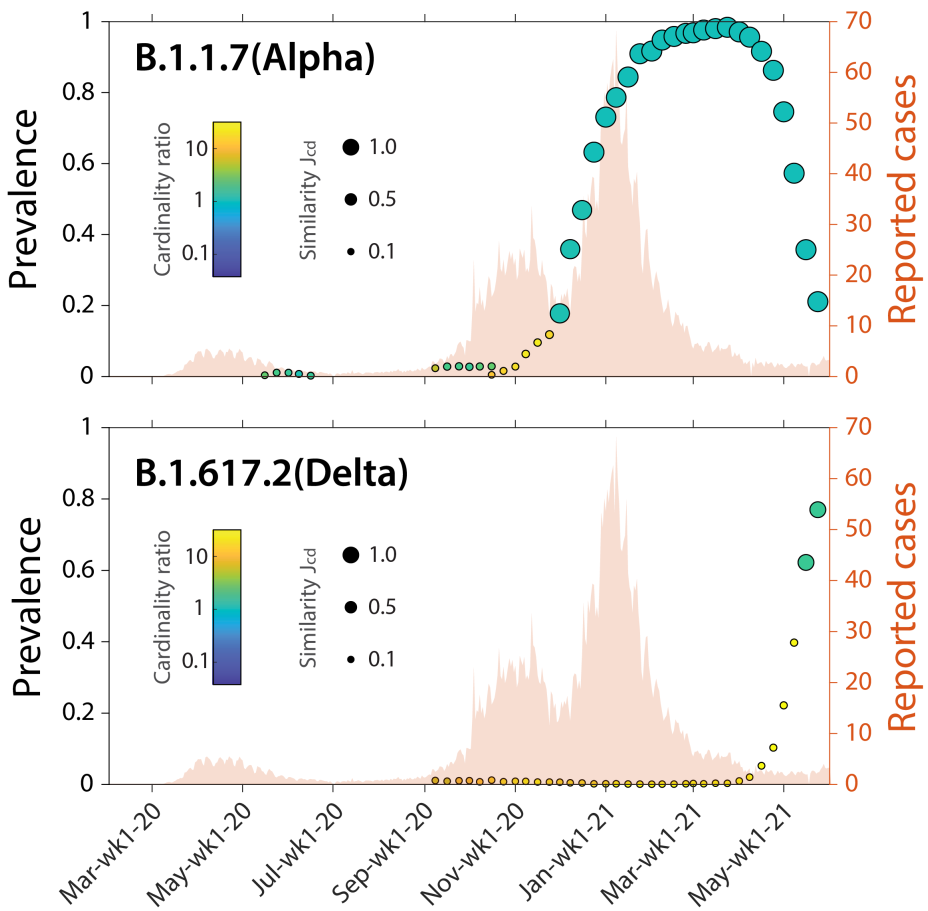


**Figure S5.** Temporal dynamics of the two variants identified in UK. We show the decline of Alpha variant occurring together with the growth of the Delta variant. Details as in panels (d–g) of Fig. 1 in the main text.
Plots were created using MATLAB R2021a (`http://www.mathworks.com`). All graphics were further processed using Adobe Illustrator 2021 (`http://www.adobe.com`).

## Supplementary Tables

**Table S1.** List of considered lineages

| WHO | Pangolin | Nextstrain | GISAID | PHE | VoC | VoI | VuI | VuM | Seq. |
|---|---|---|---|---|---|---|---|---|---|
| | B.1.1.214 | | | | - | - | - | - | 16611 |
| | B.1.1.284 | | | | - | - | - | - | 8877 |
| | B.1.1.519 | | | | - | - | - | E | 17918 |
| Alpha | B.1.1.7 | 20I/501Y.V1 | GRY | VOC-20DEC-01 | W,E,C,P | - | - | - | 776222 |
| | B.1.160.[\|20] | 20A.EU2 | | | - | - | - | - | 22240 |
| | B.1.177.[\|21\|50\|75]\|Z.1 | 20E (EU1) | | | - | - | - | - | 83587 |
| | B.1.177.12 | | | | - | - | - | - | 6549 |
| | B.1.177.[7\|8\|48\|59\|65] | | | | | | | | 9009 |
| | B.1.177.86 | | | | | | | | 5184 |
| | B.1.2 | | | | - | - | - | - | 86647 |
| | B.1.214.2 | | | | - | - | - | E | 1200 |
| | B.1.221 | 20A/S:98F | | | - | - | - | - | 12723 |
| | B.1.234 | | | | | | | | 6078 |
| | B.1.258.[\|24] | 20A/S:439K | | | - | - | - | - | 12575 |
| | B.1.258.17 | | | | | | | | 6634 |
| Beta | B.1.351 | 20H/501Y.V2 | GH/501Y.V2 | VOC-20DEC-02 | W,E,C,P | - | - | - | 19284 |
| | B.1.36.[\|8\|16\|24] | | | | | | | | 5901 |
| Epsilon | B.1.427 | 20C/S:452R | GH/452R.V1 | | C | W,E | - | - | 12889 |
| Epsilon | B.1.429 | 20C/S:452R | GH/452R.V1 | | C | W,E | - | - | 30509 |
| Eta | B.1.525 | 20A/S:484K | G/484K.V3 | VUI-21FEB-03 | - | W,E,C | P | - | 5171 |
| Iota | B.1.526 | 20C/S:484K | GH/253G.V1 | | - | W,C | - | E | 20147 |
| Iota | B.1.526.1 | 20C | | | - | C | - | E | 8201 |
| Iota | B.1.526.2 | | | | - | - | - | E | 9190 |
| | B.1.596 | | | | - | - | - | - | 9772 |
| Kappa | B.1.617.1 | 20A/S:154K | G/452R.V3 | VUI-21APR-01 | - | W,E,C | P | - | 2791 |
| Delta | B.1.617.2 | 20A/S:478K | G/478K.V1 | VOC-21APR-02 | W,E,C,P | C | - | - | 17354 |
| Lambda | C.37 | | GR/452Q.V1 | | - | W | - | E | 1083 |
| | D.2 | | | | | | | | 12335 |
| Gamma | P.1\|P.1.2 | 20J/501Y.V3 | GR/501Y.V3 | VOC-21JAN-02 | W,E,C,P | - | - | - | 22833 |
| Zeta | P.2 | 20B/S.484K | GR/484K.V2 | VUI-21JAN-01 | - | W,C | P | E | 3325 |
| | R.1 | | | | - | - | - | - | 8247 |

WHO names[16]; Pangolin lineages[13]; Nextstrain clades[14]; GISAID clades[1]; Public Health England[22] (PHE). VoC (Variant of Concern), VoI (Variant of Interest), VuI (Variant under Investigation), and VuM (Variant under Monitoring) are provided by four authoritative sources (W = WHO[16]; E = ECDC[28]; C = CDC[27]; P = PHE[22]). Numbers of sequences refer to groups of lineages that share the same dictionary (see Supplementary Table S2).

## **Table S2.** The lineage dictionary

<table>
<tr><th>Label</th><th>Dictionary: changes in > 75% sequences</th></tr>
<tr><td>B.1.1.214(JP1)</td><td>N_M234I, NSP14_P43L, NSP16_R287I</td></tr>
<tr><td>B.1.1.284(JP2)</td><td>N_P151L, NSP12_A423V, NSP3_S543P, NSP5_P108S</td></tr>
<tr><td>B.1.1.519</td><td>NSP3_P141S, NSP4_T492I, NSP6_I49V, NSP9_T35I, Spike_P681H, Spike_T478K, Spike_T732A</td></tr>
<tr><td>B.1.1.7(Alpha)</td><td>N_D3L, N_S235F, NS8_Q27*, NS8_R52I, NS8_Y73C, NSP3_A890D, NSP3_I1412T, NSP3_T183I, NSP6_F108-, NSP6_G107-, NSP6_S106-, Spike_A570D, Spike_D1118H, Spike_H69-, Spike_N501Y, Spike_P681H, Spike_S982A, Spike_T716I, Spike_V70-, Spike_Y144-</td></tr>
<tr><td>B.1.160.[|20]</td><td>N_A376T, N_M234I, NS3_Q57H, NSP12_A185S, NSP12_V776L, NSP13_E261D, NSP13_K218R, NSP4_M324I, Spike_S477N</td></tr>
<tr><td>B.1.177.12</td><td>N_A220V, N_P365S, NS3_R122I, Spike_A222V</td></tr>
<tr><td>B.1.177.[7|8|48|59|65]</td><td>N_A220V, Spike_A222V, Spike_L18F</td></tr>
<tr><td>B.1.177.86</td><td>N_A220V, NS7b_E39*, Spike_A222V, Spike_L18F</td></tr>
<tr><td>B.1.177.[|21|50|75]|Z.1</td><td>N_A220V, Spike_A222V</td></tr>
<tr><td>B.1.2(US1)</td><td>N_P199L, N_P67S, NS3_G172V, NS3_Q57H, NS8_S24L, NSP14_N129D, NSP16_R216C, NSP2_T85I, NSP5_L89F</td></tr>
<tr><td>B.1.214.2</td><td>N_D3L, NSP12_R583G, NSP3_I580V, NSP3_T1063I, NSP8_A74V, N_T205I, Spike_N450K, Spike_Q414K, Spike_T716I</td></tr>
<tr><td>B.1.221</td><td>N_P199L, NS3_G172R, NS3_Q38R, NS3_V202L, NSP3_H295Y, Spike_S98F</td></tr>
<tr><td>B.1.234</td><td>N_S194L, NSP2_V485I, NSP3_K1241R, NSP4_D217G, N_T391I</td></tr>
<tr><td>B.1.258.17</td><td>NS3_Q185H, NS8_E64*, NSP12_V720I, NSP13_A598S, NSP13_H290Y, NSP13_P53L, NSP14_E453D, NSP14_P46L, NSP3_I1683T, NSP9_M101I, Spike_H69-, Spike_L189F, Spike_N439K, Spike_V70-, Spike_V772I</td></tr>
<tr><td>B.1.258.[|24]</td><td>NSP13_H290Y, NSP3_I1683T, Spike_N439K</td></tr>
<tr><td>B.1.351(Beta)</td><td>E_P71L, NS3_Q57H, NS3_S171L, NSP2_T85I, NSP3_K837N, NSP5_K90R, NSP6_F108-, NSP6_G107-, NSP6_S106-, N_T205I, Spike_A243-, Spike_A701V, Spike_D215G, Spike_D80A, Spike_E484K, Spike_K417N, Spike_L242-, Spike_L244-, Spike_N501Y</td></tr>
<tr><td>B.1.36.[|8|16|24]</td><td>N_S194L, NS3_Q57H</td></tr>
<tr><td>B.1.427(Epsilon)</td><td>NS3_Q57H, NSP13_D260Y, NSP13_P53L, NSP2_T85I, NSP4_S395T, N_T205I, Spike_L452R, Spike_S13I, Spike_W152C</td></tr>
<tr><td>B.1.429(Epsilon)</td><td>NS3_Q57H, NSP13_D260Y, NSP2_T85I, NSP9_I65V, N_T205I, Spike_L452R, Spike_S13I, Spike_W152C</td></tr>
<tr><td>B.1.525(Eta)</td><td>E_L21F, M_I82T, N_A12G, NSP12_P323F, NSP3_T1189I, NSP6_F108-, NSP6_G107-, NSP6_S106-, N_T205I, Spike_A67V, Spike_E484K, Spike_F888L, Spike_H69-, Spike_Q52R, Spike_Q677H, Spike_V70-, Spike_Y144-</td></tr>
<tr><td>B.1.526(Iota)</td><td>N_M234I, N_P199L, NS3_P42L, NS3_Q57H, NS8_T11I, NSP13_Q88H, NSP2_T85I, NSP4_L438P, NSP6_F108-, NSP6_G107-, NSP6_S106-, Spike_A701V, Spike_D253G, Spike_E484K, Spike_L5F, Spike_T95I</td></tr>
<tr><td>B.1.526.1(Iota)</td><td>N_M234I, NS3_P42L, NS3_Q57H, NS8_A51S, NS8_T11I, NSP2_T85I, NSP4_A446V, NSP4_K399E, NSP4_L438P, NSP6_F108-, NSP6_G107-, NSP6_S106-, NSP6_V278I, N_T205I, Spike_D80G, Spike_D950H, Spike_F157S, Spike_L452R, Spike_T859N, Spike_Y144-</td></tr>
<tr><td>B.1.526.2(Iota)</td><td>N_P13L, N_S202R, NS3_P42L, NS3_Q57H, NS7a_L116F, NS8_T11I, NSP13_Q88H, NSP2_T85I, NSP3_G1128S, NSP4_L438P, NSP6_F108-, NSP6_G107-, NSP6_S106-, Spike_D253G, Spike_L5F, Spike_Q957R, Spike_S477N, Spike_T95I</td></tr>
<tr><td>B.1.596(US2)</td><td>N_P199L, N_P67S, NS3_G172V, NS3_Q57H, NS8_S24L, NSP14_N129D, NSP2_T85I, NSP5_L89F</td></tr>
<tr><td>B.1.617.1(Kappa)</td><td>N_D377Y, N_R203M, NS3_S26L, NS7a_V82A, NSP13_M429I, NSP15_K259R, NSP3_T749I, NSP6_T77A, Spike_E484Q, Spike_L452R, Spike_P681R, Spike_Q1071H</td></tr>
<tr><td>B.1.617.2(Delta)</td><td>M_I82T, N_D377Y, N_D63G, N_R203M, NS3_S26L, NS7a_T120I, NS7a_V82A, NSP12_G671S, NSP13_P77L, Spike_D950N, Spike_E156G, Spike_F157-, Spike_L452R, Spike_P681R, Spike_R158-, Spike_T19R, Spike_T478K</td></tr>
<tr><td>C.37(Lambda)</td><td>N_G214C, N_P13L, NSP3_F1569V, NSP3_P1469S, NSP3_T428I, NSP4_L438P, NSP4_T492I, NSP5_G15S, NSP6_F108-, NSP6_G107-, NSP6_S106-, Spike_D253N, Spike_F490S, Spike_G252-, Spike_G75V, Spike_L249-, Spike_L452Q, Spike_P251-, Spike_R246-, Spike_S247-, Spike_T250-, Spike_T76I, Spike_T859N, Spike_Y248-</td></tr>
<tr><td>D.2</td><td>NSP2_I120F, Spike_S477N</td></tr>
<tr><td>P.1|P.1.2(Gamma)</td><td>N_P80R, NS3_S253P, NS8_E92K, NSP13_E341D, NSP3_K977Q, NSP3_S370L, NSP6_F108-, NSP6_G107-, NSP6_S106-, Spike_D138Y, Spike_E484K, Spike_H655Y, Spike_K417T, Spike_L18F, Spike_N501Y, Spike_P26S, Spike_R190S, Spike_T1027I, Spike_T20N, Spike_V1176F</td></tr>
<tr><td>P.2(Zeta)</td><td>N_A119S, N_M234I, NSP5_L205V, NSP7_L71F, Spike_E484K, Spike_V1176F</td></tr>
<tr><td>R.1(JP3)</td><td>M_F28L, N_Q418H, N_S187L, NSP13_G439R, NSP14_P412H, Spike_E484K, Spike_G769V, Spike_W152L</td></tr>
</table>

The dictionary contains amino acid changes occurring in at least 75% of a lineage's sequences. Worldwide confounding (too frequent) changes are already removed, North-America-specific ones are not. Some lineages are grouped as they share the same dictionary of amino acid changes.

**Table S3.** Statistics on SARS-CoV-2 sequencing in world countries

| Country | #Deposited Seq. | COVID-19 Cases | Sequenced% | Delay 2020 | Delay 2021 |
|---|---|---|---|---|---|
| USA | 517,218 | 33,326,436 | 1.55 | 121.29 | 27.70 |
| United Kingdom | 425,455 | 4,515,778 | 9.42 | 37.74 | 15.65 |
| Germany | 119,813 | 3,701,692 | 3.24 | 165.17 | 21.13 |
| Denmark | 102,456 | 284,888 | 35.96 | 55.99 | 29.01 |
| Sweden | 55,158 | 1,076,993 | 5.12 | 88.45 | 46.62 |
| Japan | 49,007 | 755,711 | 6.48 | 130.27 | 50.61 |
| Switzerland | 41,853 | 696,801 | 6.01 | 91.71 | 23.09 |
| France | 39,872 | 6,104,346 | 0.65 | 143.17 | 30.22 |
| Netherlands | 34,314 | 1,684,374 | 2.04 | 61.70 | 24.56 |
| Spain | 30,510 | 3,693,012 | 0.83 | 128.99 | 36.00 |
| Italy | 29,301 | 4,225,163 | 0.69 | 139.11 | 21.65 |
| Canada | 28,000 | 1,395,336 | 2.01 | 178.37 | 73.08 |
| Belgium | 23,278 | 1,066,957 | 2.18 | 76.98 | 18.17 |
| Australia | 17,811 | 30,141 | 59.09 | 67.81 | 12.15 |
| India | 16,309 | 28,574,350 | 0.06 | 111.98 | 46.61 |
| Austria | 15,793 | 645,834 | 2.45 | 116.26 | 50.63 |
| Ireland | 14,316 | 263,252 | 5.44 | 134.10 | 21.79 |
| Poland | 13,245 | 2,874,092 | 0.46 | 109.06 | 24.32 |
| Brazil | 12,863 | 16,803,472 | 0.08 | 162.32 | 46.82 |
| Israel | 11,835 | 839,532 | 1.41 | 48.23 | 62.73 |
| Slovenia | 11,764 | 254,692 | 4.62 | 191.98 | 25.89 |
| Norway | 10,173 | 126,218 | 8.06 | 78.44 | 31.07 |
| Lithuania | 9,914 | 275,545 | 3.60 | 77.22 | 29.60 |
| Mexico | 9,517 | 2,426,822 | 0.39 | 174.72 | 29.03 |
| Portugal | 8,354 | 851,031 | 0.98 | 85.21 | 25.79 |
| Luxembourg | 7,771 | 70,088 | 11.09 | 57.58 | 30.50 |
| South Africa | 7,161 | 1,680,373 | 0.43 | 85.24 | 68.15 |
| Greece | 6,818 | 406,751 | 1.68 | 174.50 | 52.94 |
| South Korea | 6,212 | 142,851 | 4.35 | 105.06 | 33.99 |
| Finland | 5,572 | 92,913 | 6.00 | 154.45 | 63.23 |
| Iceland | 5,070 | 6,555 | 77.35 | 135.17 | 11.46 |
| Turkey | 4,820 | 4,447,074 | 0.11 | 129.20 | 27.69 |
| Philippines | 4,293 | 1,247,899 | 0.34 | 163.09 | 89.50 |
| Russia | 3,968 | 5,040,390 | 0.08 | 108.05 | 57.22 |
| Czech Republic | 3,407 | 1,662,608 | 0.20 | 141.02 | 40.82 |
| Latvia | 3,350 | 134,162 | 2.50 | 119.18 | 34.80 |
| Argentina | 3,218 | 3,884,447 | 0.08 | 196.08 | 44.64 |
| Slovakia | 2,656 | 390,129 | 0.68 | 85.22 | 23.08 |
| Singapore | 2,575 | 62,145 | 4.14 | 89.88 | 10.91 |
| Bulgaria | 2,485 | 419,180 | 0.59 | 133.08 | 40.80 |
| Qatar | 2,256 | 218,080 | 1.03 | 294.87 | 56.50 |
| Chile | 2,199 | 1,403,101 | 0.16 | 164.08 | 25.36 |
| Hong Kong | 1,867 | 11,849 | 15.76 | 125.78 | 64.61 |
| United Arab Emirates | 1,846 | 576,947 | 0.32 | 171.14 | 33.00 |
| Indonesia | 1,845 | 1,837,126 | 0.10 | 129.49 | 63.64 |
| Estonia | 1,782 | 129,909 | 1.37 | 74.25 | 33.12 |
| Croatia | 1,656 | 357,109 | 0.46 | 162.09 | 40.16 |
| Bangladesh | 1,639 | 805,980 | 0.20 | 106.80 | 33.43 |
| Thailand | 1,337 | 169,344 | 0.79 | 99.41 | 25.93 |
| Colombia | 1,334 | 3,488,046 | 0.04 | 144.99 | 40.48 |
| Peru | 1,226 | 1,965,432 | 0.06 | 166.92 | 58.72 |
| China | 1,155 | 90,697 | 1.27 | 117.00 | 16.90 |
| Reunion | 1,094 | - | - | 153.25 | 53.70 |
| New Zealand | 1,064 | 2,682 | 39.67 | 87.06 | 13.84 |

Countries contributing to the GISAID database with more than 1,000 sequences; COVID-19 cases[65]; percentage of sequences over cases; average delay (in days) between sample collection and sequence submission dates.

**Table S4.** Statistics on SARS-CoV-2 sequencing in all US states

| Country | #Deposited Seq. | COVID-19 Cases | Sequenced% | Delay 2020 | Delay 2021 |
|---|---|---|---|---|---|
| California | 58,236 | 3,689,996 | 1.58 | 99.63 | 36.42 |
| Texas | 56,688 | 2,880,056 | 1.97 | 152.86 | 32.36 |
| New York | 42,174 | 1,151,994 | 3.66 | 102.36 | 23.35 |
| Florida | 31,293 | 2,286,332 | 1.37 | 97.15 | 23.60 |
| Michigan | 25,742 | 994,774 | 2.59 | 98.55 | 24.51 |
| Minnesota | 22,624 | 600,990 | 3.76 | 84.64 | 24.19 |
| Washington | 22,522 | 438,544 | 5.14 | 66.34 | 22.27 |
| Massachusetts | 18,109 | 705,818 | 2.57 | 149.46 | 20.34 |
| Illinois | 17,143 | 1,383,739 | 1.24 | 190.38 | 25.13 |
| Arizona | 16,661 | 882,691 | 1.89 | 165.14 | 30.43 |
| Wisconsin | 15,056 | 674,939 | 2.23 | 86.00 | 24.55 |
| Colorado | 14,791 | 545,002 | 2.71 | 137.16 | 27.98 |
| Pennsylvania | 14,229 | 1,204,099 | 1.18 | 132.46 | 22.64 |
| New Jersey | 13,069 | 1,026,054 | 1.27 | 188.05 | 27.03 |
| Maryland | 12,155 | 460,339 | 2.64 | 77.58 | 24.73 |
| Utah | 12,115 | 406,825 | 2.98 | 117.14 | 71.36 |
| North Carolina | 9,130 | 1,004,622 | 0.91 | 157.64 | 24.33 |
| Virginia | 7,827 | 676,300 | 1.16 | 75.40 | 25.31 |
| Wyoming | 7,425 | 60,549 | 12.26 | 133.69 | 29.24 |
| Oregon | 7,413 | 202,247 | 3.67 | 102.72 | 38.63 |
| Georgia | 7,403 | 1,125,017 | 0.66 | 121.46 | 27.62 |
| Connecticut | 7,370 | 347,748 | 2.12 | 91.18 | 22.28 |
| Indiana | 6,435 | 745,690 | 0.86 | 217.37 | 26.65 |
| Ohio | 6,326 | 1,103,380 | 0.57 | 171.57 | 34.22 |
| New Mexico | 5,152 | 203,330 | 2.53 | 97.07 | 29.94 |
| Louisiana | 5,032 | 472,617 | 1.06 | 90.36 | 27.05 |
| West Virginia | 4,977 | 162,111 | 3.07 | 136.66 | 35.41 |
| Tennessee | 4,084 | 863,890 | 0.47 | 145.73 | 27.36 |
| Nevada | 3,914 | 325,031 | 1.20 | 140.95 | 27.29 |
| Maine | 3,456 | 68,057 | 5.08 | 75.98 | 20.94 |
| Rhode Island | 3,434 | 151,953 | 2.26 | 122.11 | 23.60 |
| Missouri | 3,123 | 601,503 | 0.52 | 212.70 | 27.48 |
| Kansas | 2,971 | 314,689 | 0.94 | 195.82 | 16.79 |
| South Carolina | 2,949 | 593,061 | 0.50 | 122.58 | 31.48 |
| Alabama | 2,695 | 546,259 | 0.49 | 74.46 | 24.67 |
| Hawaii | 2,206 | 34,568 | 6.38 | 104.46 | 30.31 |
| New Hampshire | 2,065 | 98,840 | 2.09 | 110.76 | 20.58 |
| Kentucky | 1,954 | 459,540 | 0.43 | 152.10 | 25.06 |
| Nebraska | 1,944 | 223,517 | 0.87 | 98.05 | 27.12 |
| Alaska | 1,908 | 67,591 | 2.82 | 72.98 | 20.51 |
| Delaware | 1,784 | 108,957 | 1.64 | 120.17 | 24.36 |
| North Dakota | 1,715 | 110,151 | 1.56 | 160.14 | 36.85 |
| Idaho | 1,149 | 192,630 | 0.60 | 147.05 | 27.03 |
| Vermont | 1,040 | 24,240 | 4.29 | 132.83 | 19.29 |
| Iowa | 936 | 371,761 | 0.25 | 97.77 | 28.34 |
| Mississippi | 903 | 317,783 | 0.28 | 116.04 | 24.59 |
| Arkansas | 787 | 341,889 | 0.23 | 86.97 | 30.50 |
| Montana | 766 | 112,057 | 0.68 | 135.32 | 23.13 |
| Oklahoma | 693 | 453,552 | 0.15 | 108.99 | 27.31 |
| South Dakota | 561 | 124,242 | 0.45 | 123.46 | 27.42 |

All 50 US states contributing to the GISAID database; COVID-19 cases[66]; other columns as in Supplementary Table S3.

**Table S5.** Size of the aggregated data matrices

| Country/US State | #rows (changes) | #columns (weeks) |
|---|---|---|
| United Kingdom | 3808 | 64 |
| Germany | 2137 | 64 |
| Denmark | 1456 | 59 |
| California | 1278 | 62 |
| Switzerland | 1011 | 62 |
| Texas | 1009 | 59 |
| New York | 929 | 61 |
| Sweden | 860 | 61 |
| France | 841 | 62 |
| Florida | 773 | 61 |
| Italy | 635 | 63 |
| Netherlands | 626 | 62 |
| Spain | 620 | 62 |
| Washington | 616 | 61 |
| Japan | 594 | 63 |
| Canada | 583 | 56 |
| Belgium | 551 | 61 |
| Massachusetts | 517 | 58 |
| Minnesota | 506 | 58 |
| Michigan | 474 | 58 |
| Illinois | 415 | 60 |
| Pennsylvania | 406 | 56 |
| Colorado | 404 | 54 |
| Ireland | 371 | 55 |
| Arizona | 365 | 58 |
| Maryland | 347 | 46 |
| Wisconsin | 344 | 60 |
| New Jersey | 336 | 38 |
| Utah | 324 | 57 |
| India | 306 | 59 |
| North Carolina | 304 | 54 |
| Oregon | 273 | 57 |
| Connecticut | 266 | 42 |
| Ohio | 245 | 40 |
| Virginia | 239 | 59 |
| Georgia | 238 | 54 |
| Australia | 225 | 62 |
| Indiana | 224 | 28 |
| Wyoming | 203 | 42 |
| New Mexico | 185 | 52 |
| Brazil | 171 | 59 |
| Louisiana | 158 | 58 |
| Austria | 140 | 53 |
| South Africa | 84 | 56 |

Matrices are extracted from GISAID for the countries and US states analyzed in this study, ordered by number of rows (changes).
